# NEDD9 Restrains dsDNA Damage Response during Non-Small Cell Lung Cancer (NSCLC) Progression

**DOI:** 10.3390/cancers14102517

**Published:** 2022-05-20

**Authors:** Mariya Tikhomirova, Iuliia Topchu, Aleksandra Mazitova, Vitaly Barmin, Ekaterina Ratner, Alexey Sabirov, Zinaida Abramova, Alexander Y. Deneka

**Affiliations:** 1 Institute of Fundamental Medicine and Biology, Kazan Federal University, 420000 Kazan, Russia; mvtikhomirova@kpfu.ru (M.T.); iuliia.topchu@northwestern.edu (I.T.); aleksandra.mazitova@cshs.org (A.M.); zinaida.abramova@kpfu.ru (Z.A.); 2 Feinberg School of Medicine, Northwestern University, Chicago, IL 60610, USA; 3 Department of Medicine and Biomedical Science, Cedars-Sinai Medical Center, Los Angeles, CA 90048, USA; 4 Moscow P.A. Gertsen Oncological Research Institute, 125284 Moscow, Russia; vitaly.barmin@gmail.com; 5 Republican M.Z.Sigal Clinical Oncology Hospital, 420029 Kazan, Russia; katyratner@gmail.com (E.R.); a-sabirov@yandex.ru (A.S.); 6 Program in Molecular Therapeutics, Fox Chase Cancer Center, Philadelphia, PA 19111, USA

**Keywords:** HEF1, DNA damage response, CHK2, ERCC4, non-small cell lung cancer, overall survival

## Abstract

**Simple Summary:**

Altered DNA damage response (DDR) contributes to numerous processes during the progression of tumors, such as genomic instability, the emergence of neoantigens, aberrations in cell-cell signaling, and acquired tumor resistance to DNA damaging agents, such as platinating agents and irradiation. This study describes a novel role for the scaffolding protein NEDD9 in regulating DDR signaling and characterizes its effects on sensitivity to DNA damaging therapies in a non-small cell lung cancer (NSCLC) setting. Our data demonstrate that NEDD9 depletion is capable of upregulating ATM-CHK2 signaling, shifting NEDD9 depleted cells towards a more mesenchymal phenotype and elevated sensitivity to UV irradiation. Immunohistochemical analysis of the cohort of human NSCLC samples revealed an association between reduced NEDD9 protein expression and a decrease in overall (OS) survival of NSCLC patients.

**Abstract:**

DNA damaging modalities are the backbone of treatments for non-small cell lung cancer (NSCLC). Alterations in DNA damage response (DDR) in tumor cells commonly contribute to emerging resistance to platinating agents, other targeted therapies, and radiation. The goal of this study is to identify the previously unreported role of NEDD9 scaffolding protein in controlling DDR processes and sensitivity to DNA damaging therapies. Using a siRNA-mediated approach to deplete NEDD9 in a group of human and murine *KRAS/TP53*-mutant NSCLC cell lines, coupled with a set of cell viability and clonogenic assays, flow cytometry analysis, and Western blotting, we evaluated the effects of NEDD9 silencing on cellular proliferation, DDR and epithelial-to-mesenchymal transition (EMT) signaling, cell cycle, and sensitivity to cisplatin and UV irradiation. Using publicly available NSCLC datasets (TCGA) and an independent cohort of primary NSCLC tumors, subsequent in silico and immunohistochemical (IHC) analyses were performed to assess relevant changes in NEDD9 RNA and protein expression across different stages of NSCLC. The results of our study demonstrate that NEDD9 depletion is associated with the increased tumorigenic capacity of NSCLC cells. These phenotypes were accompanied by significantly upregulated ATM-CHK2 signaling, shifting towards a more mesenchymal phenotype in NEDD9 depleted cells and elevated sensitivity to UV-irradiation. IHC analyses revealed an association between reduced NEDD9 protein expression and a decrease in overall (OS) and progression-free survival (PFS) of the NSCLC patients. These data, for the first time, identified NEDD9 as a negative regulator of ATM kinase activity and related DDR signaling in numerous *KRAS/TP53* mutated NSCLC, with its effects on the regulation of DDR-dependent EMT signaling, sensitivity to DNA damaging modalities in tumor cells, and the survival of the patients.

## 1. Introduction

According to the American Cancer Society, 228,820 new cases of lung cancer were diagnosed in 2020, with nearly 136,000 deaths [1]. Lung cancer has the lowest five-year survival rate (18.6%) compared to other leading cancers [1]. Given that very few lung tumors (~16%) are detected at an early stage, the five-year survival rate for patients with metastases is only 5% [2]. A common feature of non-small cell lung cancer (NSCLC), the most common histological type of lung cancer, is the presence of somatic activating mutations of *KRAS* (~30%) accompanied by inactivating mutations of the tumor suppressor *TP53* [3,4], leading to activation of pro-proliferative signaling and disruption of G1/S checkpoint machinery, lack of cell cycle arrest in response to DNA-damaging therapies, such as platinating agents (cisplatin, carboplatin) and irradiation: the commonly used treatment approaches for NSCLC without targetable driver mutations [5,6]. 

Several studies demonstrated that DNA damage promotes chemoresistance and drives epithelial-to-mesenchymal transition (EMT) via the activation of altered ataxia telangiectasia mutated (ATM) dependent signaling in several cancer settings, including NSCLC [7,8]. ATM and its partner ATR belong to the class-IV phosphoinositide 3-kinase (PI3K)-related kinase (PIKK) family [9] and mediate DNA damage response (DDR) by activating checkpoint kinases CHK1 and CHK2. Activated ATM and ATR kinases are capable to induce cell cycle arrest and DNA repair in response to different types of DNA lesions. ATM responds primarily to DNA double-strand breaks (DSBs), to which it is recruited through specific co-factors [10], while ATR responds to single-stranded DNA breaks and keeps integrity of replicating chromosomes [11]. However, recent studies demonstrate that ATM kinase can be involved in cellular responses to processes not related to DNA repair, such as cellular homeostasis [12], response to oxidative stress [13], and cell cycle regulation, when activated in the absence of DNA damage by Aurora-B kinase during cell cycle progression [14], and others. 

NEDD9 (neural precursor cell expressed, developmentally downregulated 9, also known as Cas-L and HEF1) is a scaffolding protein implicated in intracellular signaling processes that regulate attachment, migration [15,16], cell survival [17], mitogenic signaling, and cell cycle control [18,19,20,21]. Altered NEDD9 expression accompanies and promotes metastasis in a large number of cancers, including NSCLC. Genetic ablation of NEDD9 results in significant signaling changes during tumor initiation and progression [22,23,24]. Both overexpression and loss of NEDD9 function have been found to be tumor-promoting in different cancers, likely because either form of disruption of its role as a scaffold impairs downstream processes. Importantly, several papers suggest direct interaction of NEDD9 with signaling proteins that are involved in regulation of cell cycle progression. NEDD9 binds to Aurora-A kinase (AURKA) and is required to support its activity. The loss of this interaction induces centrosomal defects, leads to accumulation of mitotic abnormalities and aneuploidy [25,26]. 

Based on these and other findings, and given that cell cycle regulation processes are highly dependent on the integrity of DNA, we have hypothesized that NEDD9 scaffolding protein might play a role in the control of DNA damage response and can potentially affect the sensitivity of DNA damaging therapies, particularly in the context of NSCLC. Today, there are no published studies in the literature that have addressed a potential link between NEDD9 and cellular dependence on DDR. The results reported here indicate a striking modifier function of NEDD9 on the activity of ATM kinase and related signaling in *KRAS/TP53* mutated NSCLC, with its effects on the regulation of both DDR and EMT processes, sensitivity to DNA damaging modalities, and the survival of the patients. 

## 2. Materials and Methods

### 2.1. Cell Culture and Transient Transfection

The A549, H460, and H1299 human NSCLC cell lines were obtained from the American Type Culture Collection (ATCC), and their identity was verified by STR profiling. Human cell lines were selected based on the significant expression of NEDD9 and driver mutations resembling the heterogeneity of NSCLC, as determined by the analysis of data available in the cancer cell line encyclopedia (CCLE) at cBioPortal for Cancer Genomics (http://www.cbioportal.org/) (accessed on 30 April 2021) [27]: A549 and H460 cell lines have *KRAS* mutations and wt *TP53*, while H1299 cell line has *NRAS* mutation and *TP53* deletion. The murine cell lines (344SQ, 531LN2) were derived from *Kras^LA1/+^* /*Trp53^R172HΔg/+^* mice [28]. A549 cells were cultured in DMEM media. All other cells were cultured in RPMI-1640 media, containing 10% fetal bovine serum (FBS), L-glutamine (L-glu), and penicillin/streptomycin (pen/strep).

For the purpose of transient transfection, NEDD9 Smartpool siRNA (with four pre-mixed siRNAs, cat#M-019466-02, target sequences 5′-AGGAACUGGCCUUUCGCAA-3′; 5′-CUACCAAAAUCAGGGAAUU-3′; 5′-CCUCUGGACUGAUGCAGCA-3′; 5′-CCAAGAACAAGAGGUAUAU-3′) was purchased from Dharmacon (Lafayette, CO, USA). Scrambled negative control siRNA (cat# AM4635) was purchased from Ambion (Thermo Fisher Scientific, Foster City, CA, USA). NSCLC cells were plated in 6-well plates at 20% confluence. Transfection was performed with siRNAs (final concentrations of 30 nM) using Lipofectamine RNAiMAX (Thermo Fisher Scientific, Foster City, CA, USA) transfection reagent according to the manufacturer’s instructions. The cells were processed 48 h post-transfection for further analysis unless another timeframe is indicated.

### 2.2. Cell Viability Assays and Determination of Cisplatin Effective Dose

To analyze the effects of different growing conditions on proliferation, cells (2000 cells/well) were plated in 96-well cell culture plates in a complete medium. After 24 h, serum-free, glucose- and glutamine-depleted medium was applied, and viability assay measurements were performed 4 days after the application using resazurin reagent (cat# R7017, Sigma-Aldrich, St. Louis, MO, USA) according to the manufacturer’s protocols. 

For the determination of the optimal effective concentration of cisplatin, the IC50 concentration was calculated for each cell line. Briefly, 2000 cells/well were plated in 96-well cell culture plates in complete media, and cisplatin was added using an Xplorer^®^ Vario single-channel electronic pipettor (Eppendorf, Hamburg, Germany) in a range from 0 to 10 uM. The dose-response curves and IC50 values were calculated using GraphPad Prism 7 (San Diego, CA, USA) using the multiple dose-response model function. The concentration of ~IC30 (500 nM) was selected for further experiments to minimize possible off-target effects. All assays were performed in 3 technical repeats and 3 biological repeats. 

### 2.3. Cell Migration (Wound Healing) Assays

Cell migration was evaluated by a scratch wound assay. The cells were seeded at a concentration of 12 × 10^4^ cells/well in 6-well plates and with SCR control or siNEDD9 siRNAs for 24 h. “Wound” scratch was performed 24 h post-transfection. In order to avoid cell proliferation during cell migration, transfection was performed in serum-starved conditions. A scratch was created with a 10 μL plastic pipette tip on the cell monolayer. The images of the scratch wounds were captured using an AMG Evos FL microscope (Thermo Fisher Scientific, Foster City, CA, USA) at 0, 3, 6, and 24 h after scratching. The migration is represented as percentage of wound closure between the time zero and respective timepoint. Area measurements were done using NIH ImageJ Imaging Software (Rayne Rasband, National Institutes of Health, Bethesda, MD, USA). Assay was performed in 3 biological repeats.

### 2.4. Clonogenic Survival Assays and UV Irradiation Experiments

48 h post siRNA transfection, 500–1000 cells were plated in 6-well plates and incubated in complete media for 12 days. For one arm of the study, 24 h post-seeding cells were irradiated using a germicidal lamp (254 nm) at a dose rate of 5–10 J/m2 as measured with a radiometer VLX-3W (Vilber, Seoul, Korea). For another arm of the study, 500 nM cisplatin or vehicle were added. The medium was replaced every three days, along with cisplatin replenishment. 10% acetic acid/10% methanol solution was used to fix the cells. Staining was done using 0.5% (*w*/*v*) crystal violet. A colony was defined as consisting of >50 cells and was counted using ImageJ software (Rayne Rasband, National Institutes of Health, Bethesda, MD, USA), as described previously [29].

### 2.5. Flow Cytometry Assays for Cell Cycle Analysis

The cells were treated with indicated siRNAs and vehicle or cisplatin for 48 h (si-RNAs) and 6 h (cisplatin), then stained with PI/RNAse staining buffer (BD Biosciences, San Jose, CA, USA). The cell cycle distribution was acquired by flow cytometry on the BD LSRII Flow cytometer and analyzed with FlowJo software (FlowJo LLC, Ashland, OR, USA).

### 2.6. Immunofluorescent Detection of γ-H2AX

To assess the levels of γ-H2AX foci cells were plated in triplicates in 96-well plates and treated with vehicle or cisplatin in the concentrations indicated. Treatment started 24 h after plating. After 72 h of incubation, the cells were fixed and stained with anti-γ-H2AX primary antibody (1:1000, Mouse Monoclonal, Millipore Upstate, Billerica, MA, USA), and FITC-tagged secondary antibody (1:1000, goat anti-mouse IgG (H + L), Alexa Fluor^®^ 488, Thermo Scientific, Waltham, MA, USA), and counterstained with Hoechst 33342 nuclear stain (H3570, Thermo Scientific, Waltham, MA, USA). Cells were imaged using a confocal microscope and scored using ImageXpress software as previously described [30].

### 2.7. Western Blot Analysis

The cells were lysed in Pierce^TM^ RIPA Buffer (ThermoScientific, Waltham, MA, USA). The protein concentrations were established using the Pierce BCA Protein Assay Kit (Thermo Scientific, Waltham, MA, USA). Western blotting was performed using standard procedures, and the blots were developed using Clarity Western ECL Substrate (Bio-Rad Laboratories, Hercules, CA, USA). Original images of western blot added to the Supplementary Data. Primary antibodies used were as follows: ERCC4 (#13465), p-ATM (Ser1981) (#13050), p-ATR (Ser428) (#2853), p-Chk1 (Ser345) (#2341), p-Chk2 (Thr68) (#2197), p-CDC25C (Ser216) (#9528), p-H2AX (Ser139) (#3257000) (1:1000; Cell Signaling Technology, Danvers, MA, USA); NEDD9 (ab18056, 1:1000), beta-Actin (ab49900, 1:100,000) (Abcam; Cambridge, MA, USA), Vinculin (700062) (1:1000, Invitrogen, Thermo Fisher Scientific, Waltham, MA, USA). Vinculin and beta-Actin were used as the loading controls. The quantification was performed using NIH ImageJ Imaging Software (Rayne Rasband, National Institutes of Health, USA).

### 2.8. Immunohistochemistry and Human Samples Cohort

In the current retrospective study, we used randomly selected formalin-fixed paraffin-embedded (FFPE) NSCLC primary tumor samples (*n* = 16) collected from the Republican M.Z. Sigal Clinical Oncology Hospital’s Repository, Kazan, Russia. The study was conducted in accordance with the Declaration of Helsinki (as revised in 2013). This study was approved by the local ethics committee of the Republican M.Z. Sigal Clinical Oncology Hospital. The patients underwent histopathological verification for the presence of tumors, and the Institutional Review Board (IRB) approved the informed consent for storing tissue and the reviewing of de-identified clinical data (IRB No. 8, 13 February 2018). Clinical information (Supplementary Materials Table S1) was abstracted from the Repository database in an anonymized fashion, and all of the samples were de-identified.

Immunohistochemical (IHC) staining was performed using standard methods after morphological evaluation of hematoxylin and eosin (H&E) stained sections. First, 3–5 μm formalin-fixed, paraffin-embedded sections were deparaffinized in 100% xylenes 3 times for 5 min each, followed by dehydration in a sequence of ethanol solutions (100%, 70%, and 50%) for 3 min each. Heat-induced epitope retrieval was performed in 0.01 M citrate buffer (pH 6.0). Slides were stained with NEDD9 (ab18056 2G9, Abcam, Cambridge, MA, USA) primary antibody and Mouse and Rabbit Specific HRP/DAB IHC Detection kit–Micro-polymer (Abcam ab236466, Cambridge, MA, USA) according to manufacturer’s instructions. Rabbit IgG antibody (I-1000, Vector Laboratories, Burlingame, CA, USA) was used as a negative control to confirm the absence of specific staining. Next, sections were counter-stained with hematoxylin. In the end, all of the sections were dehydrated in a sequence of ethanol solutions (70%, 96%, and 100%) and xylene for 30 s, respectively. Coverslips were mounted using Histomount histological mounting medium (HS-103, National Diagnostics, Atlanta, GA, USA).

### 2.9. The Cancer Genome Atlas (TCGA) Analysis

For The Cancer Genome Atlas (TCGA) analysis, the datasets for NSCLC were accessed and analyzed using tools available at cBioPortal for Cancer Genomics (http://www.cbioportal.org/) (accessed on 31 December 2021) [27].

### 2.10. Statistical Analysis

Mann–Whitney U two-tailed tests were used for pairwise comparisons (all assumptions for the test were met). The difference between the patient cohorts was compared using a log-rank test. *p*-values < 0.05 were considered statistically significant, and the data were presented as mean and S.E.M. The Kaplan–Meier method was used to plot survival curves, as well as to calculate median survival. All data were processed using Microsoft Excel and GraphPad Prism 7 (GraphPad Software, San Diego, CA, USA) software. 

## 3. Results

### 3.1. NEDD9 Depletion Is Associated with Increased Tumorigenic Capacity in Human and Murine NSCLC Cell Line Models

**Figure 1:**
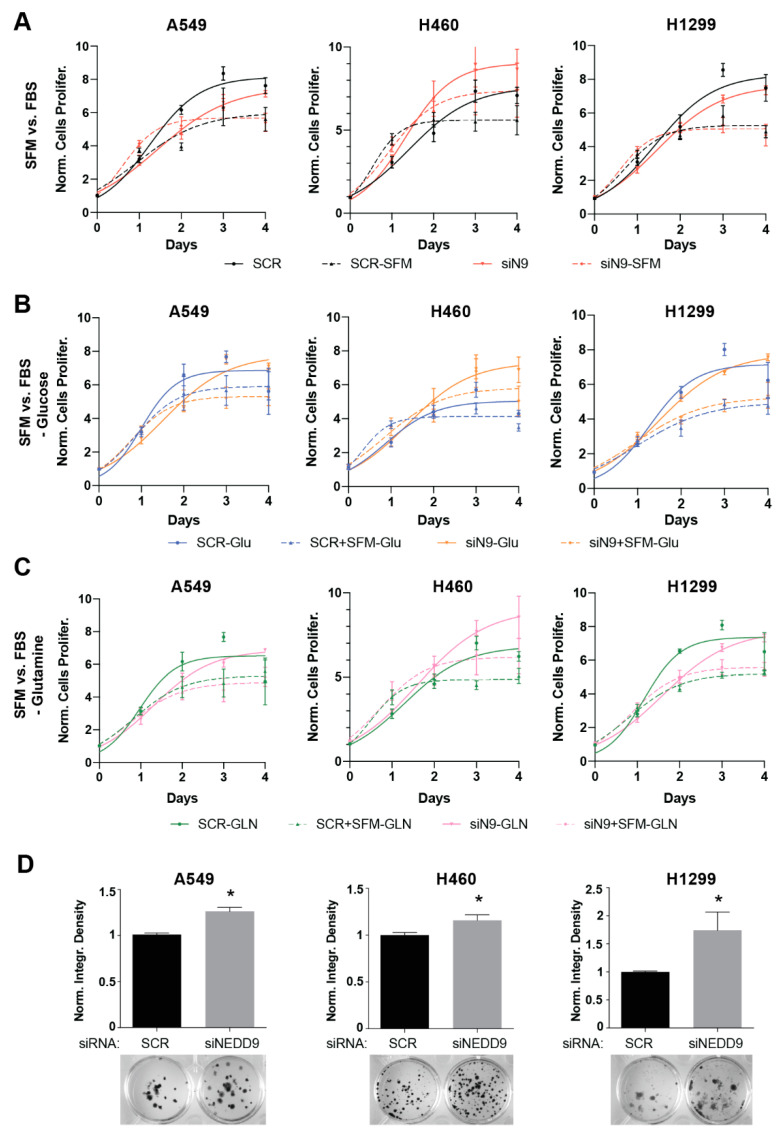
Transient NEDD9 loss promotes long term NSCLC cell survival: (**A**–**C**) Quantification of resazurin reagent-based viability assays in three human NSCLC cell lines after transfection with siRNA targeting NEDD9 or scrambled control in serum-free (SFM, dashed line) vs. serum rich (FBS, solid line) (**A**) conditions; and with additional depletion of glucose (**B**) and glutamine (**C**). (**D**) Quantification and representative images for clonogenic growth, 12 days after treatment of ×3 NSCLC cell lines treated with siNEDD9 or SCR control siRNAs. All graphs: *, *p* < 0.05, versus SCR control.

To establish whether changes in NEDD9 expression are capable of influencing the cellular capacity for replication and growth in an NSCLC setting, we directly assessed the functional consequence of NEDD9 transient depletion (Figure S1A) on the proliferative capacity of NSCLC cells (Figure 1). Intriguingly, NEDD9-depleted cells grew comparably to SCR transfected control cells, whether in rich media or in serum-free media that more closely simulated nutrient-poor conditions in tumors (Figure 1A) or in serum-free medium with additional glucose depletion (Figure 1B). NEDD9-depleted cells also proliferated comparably to SCR-transfected control cells in a medium depleted of serum and glutamine (Figure 1C), excluding the possibility that starved cells had become dependent on glutaminolysis as an alternative source of ATP [31]. Altogether, these results differ from other published studies, suggesting that siRNA transient NEDD9 depletion induces distinct cellular signaling alterations specific to the NSCLC setting.

To assess the consequences of NEDD9 depletion on the long-term replication capacity of the cells, we next performed clonogenic assays in three independent human and two murine NSCLC cell lines (Figure 1D and Figure S1B). The cells were either transfected with SCR non-targeting siRNA or siNEDD9 smartpool siRNA and grown over the course of 12 days. In this assay, the impact of the NEDD9 depletion on viability was extremely pronounced, resulting in 1.5 to 2 times greater colony formation in all five NEDD9-depleted NSCLC cell lines versus the SCR-transfected cells. Interestingly, cells with low NEDD9 remained sensitive to the treatment with cisplatin, which was effective in ameliorating the pro-proliferative effect of NEDD9 depletion (Figure S1C,D).

Altogether, these data suggest that transient NEDD9 depletion itself is capable of inducing the clonogenic capacity of murine and human NSCLC cells and promoting growth in long-term settings.

### 3.2. Upregulation of ATM Is Notable in NEDD9 Depleted and Short-Time Cisplatin Treated NSCLC Cells

**Figure 2:**
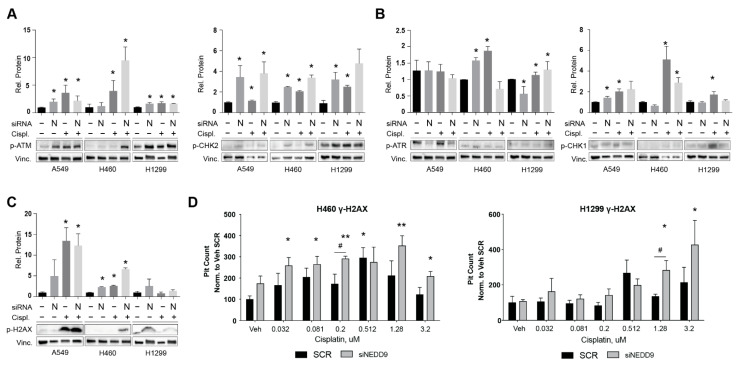
Activation of DNA damage response signaling in NEDD9 depleted NSCLC cells: (**A**–**C)** Quantification (top) and representative Western blot images (bottom) for indicated proteins in human NSCLC cell lines after 48 h transfection with siNEDD9 or SCR control siRNAs and 6 h of treatment with cisplatin or vehicle control. (**D**) γH2AX-positive foci in H460 and H1299 cell lines, 72 h after treatment with indicated siRNAs and vehicle or cisplatin, as indicated. All graphs: *, *p* ≤ 0.05; **, *p* ≤ 0.01 relative to SCR vehicle controls; #, *p* ≤ 0.05, SCR vs siNEDD9.

To gain insight into the mechanism by which NEDD9 depletion resulted in elevated NSCLC proliferation, we considered the fact that KRAS-TP53mut-driven NSCLC is dependent on altered DNA damage response (DDR) machinery [8,32,33]. To investigate the effects of transient NEDD9 depletion on DDR machinery, we siRNA-depleted *NEDD9* expression in the A549, H460, and H1299 NSCLC cell lines for 48 h, with an additional cisplatin treatment (500 nM, 6 h) benchmark, and then probed the expression and activity of the single- and double-stranded DNA damage response associated proteins using Western blotting. After 48 h, the depletion of NEDD9 caused the activation of the ds-DDR marker p-Ser^1981^ ATM [34], with statistically significant magnitude in two out of three human cell lines and statistically significant activation of its downstream mediator p-Thr^68^ CHK2 in all three cell lines (Figure 2A). The treatment with cisplatin resulted in a comparable statistically significant pattern of ATM-CHK2 axis activation in SCR vs. siNEDD9 transfected cells, with additionally elevated CHK2 activation in siNEDD9 cells versus the vehicle treated cells, suggesting that the depletion of NEDD9 is capable of inducing ATM-mediated ds-DDR independently and additively to cisplatin. Alternatively, NEDD9 depletion did not result in the strong activation of the ss-DDR marker p-Ser^428^ ATR [34] and its downstream mediator p-Ser^345^ CHK1, suggesting that this pathway was not affected by NEDD9 depletion (Figure 2B). In addition, the depletion of NEDD9 very significantly, and to an extent comparable with cisplatin, induced the phosphorylation of γH2AX, a phosphorylated histone mark associated with unrepaired DNA breaks [35]. This was assessed by Western blotting (Figure 2C) and high throughput automated quantification of γH2AX foci (Figure 2D). These data indicate that NEDD9 silencing is capable of potentiating foci accumulation versus SCR-transfected cells treated with cisplatin, reflecting the greater induction of a DNA damage response, in agreement with previous reports [36]. 

### 3.3. NEDD9-Mediated ATM Activation Is Associated with Elevated Epithelial to Mesenchymal Transition (EMT) Rather Than Cell Cycle Alterations in NSCLC Cells

**Figure 3:**
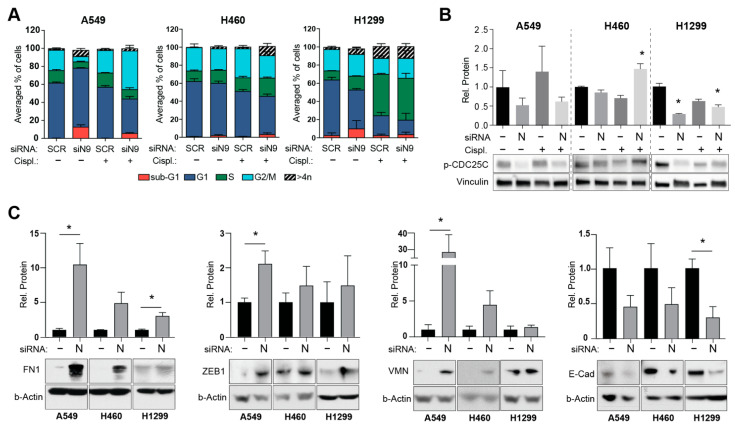
NEDD9 depletion is associated with EMT activation in NSCLC cell lines: (**A**) Quantification of cell cycle compartmentalization changes, averaged from three repetitions of experiments in A549, H460, and H1299 NSCLC cell lines, after 48 h transfection with siNEDD9 or SCR control siRNAs and 6 h of treatment with cisplatin or vehicle control. (**B**) Quantification (top) and representative Western blot images (bottom) for p-CDC25C in human NSCLC cell lines after 48 hours transfection with siNEDD9 or SCR control siRNAs and 6 h of treatment with cisplatin (500 nM) or vehicle control. (**C**) Quantification (top) and representative Western blot images (bottom) for EMT markers in SCR vs siNEDD9 depleted NSCLC cell lines. All graphs: *, *p* ≤ 0.05 relative to SCR control.

ATM/ATR downstream kinases CHK1 and CHK2 negatively regulate entry into the M phase, serving as a G2/M checkpoint [37], resulting in cell cycle arrest to repair ongoing DNA damage. To address this possibility and assess the alterations in the cell cycle, we performed FACS analysis in SCR and siNEDD9-depleted human NSCLC cells, treated with vehicle or cisplatin (Figure 3A and Figure S2). The cisplatin treatment resulted in a greater accumulation of G2/M cells compared to the vehicle, with an additionally notable S phase arrest in H1299 cells; however, the cells with depleted NEDD9 did not present with cell cycle alterations different from those in SCR transfected cells, both in the vehicle and cisplatin treatment conditions, suggesting that elevated ATM-CHK2 signaling was not associated with a cell cycle arrest in the NEDD9 depleted setting. Additionally, Western blot analysis demonstrated that the silencing of NEDD9 did not result in the enhanced activation of p-Ser^216^ CDC25C kinase (Figure 3B), a cell cycle checkpoint kinase downstream of the CHK1 and CHK2 signaling pathways [34]. 

Considering the reported connection between elevated ATM signaling and changes in epithelial to mesenchymal transition (EMT) [8] and known associations between altered NEDD9 expression levels and EMT [23,38,39], we performed several analyses to assess potential changes in the EMT-related markers in our NSCLC cell lines upon siNEDD9 depletion. Using the Western blot approach, we detected significantly increased expression of mesenchymal markers fibronectin 1 (FN1), ZEB1, and vimentin (VMN) in siNEDD9-depleted cells compared to the SCR-transfected cells and decreased levels of epithelial marker E-cadherin (Figure 3C). Additionally, we performed a wound-healing assay to test if these mesenchymal phenotypes were associated with the increased migration activity of NEDD9-depleted NSCLC cells. The results did not demonstrate any statistically significant difference in wound-healing speed between SCR and siNEDD9-transfected cells, although we noted the presence of numerous rounded “amoeboid” cells along the scratch border after NEDD9 depletion (Figure S3). These observations align with the published literature, which reports that NEDD9 is required for ROCK-dependent signaling to drive elongated, mesenchymal-type invasion [40,41].

Altogether, our data suggest that depletion of NEDD9 is capable of inducing processes of EMT, resulting in a more mesenchymal phenotype, which is associated with activation of non-canonical ATM signaling in NSCLC cells.

### 3.4. NEDD9 Depletion Is Associated with Increased Sensitivity to UV-Induced dsDNA Breaks

**Figure 4:**
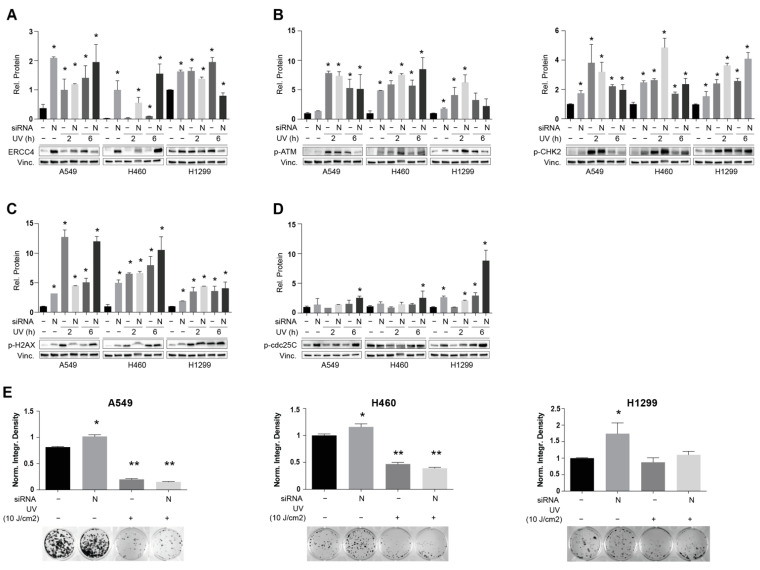
NEDD9 depletion is associated with increased sensitivity to UV-induced dsDNA breaks: (**A**–**D**) Quantification (top) and representative Western blot images (bottom) for indicated proteins in human NSCLC cell lines after 48 h transfection with siNEDD9 or SCR control siRNAs and UV irradiation as indicated. (**E**) Quantification and representative images for clonogenic growth, 12 days after treatment of ×3 human NSCLC cell lines with siNEDD9 or SCR control siRNAs and UV irradiation as indicated. All graphs: *, *p* ≤ 0.05; **, *p* ≤ 0.01 relative to SCR non-irradiated controls.

Next, we investigated potential candidate effectors related to DDR that might be mediated by the action of NEDD9. Intriguingly, we determined that NEDD9 inhibition results in the statistically significant elevation of ERCC4 expression levels in three human and two murine NSCLC cell lines (Figure 4A and Figure S4A). The ERCC4 gene is involved in nucleotide excision repair; a DDR commonly activated after dsDNA brakes caused by exposure to ultraviolet (UV) irradiation [42]. The mechanistic analysis of human and murine NSCLC cell lines demonstrated that UV irradiation for 2 and 6 h of SCR and siNEDD9-depleted cells resulted in even greater ERCC4 activation upon NEDD9 depletion in A549 and H460 cell lines (Figure 4A and Figure S4A) and significant activation of ATM-CHK2 signaling axis in both SCR and siNEDD9-treated cells versus the SCR non-irradiated control in four out of five cell lines (Figure 4B and Figure S4B). These observations were accompanied by the elevated activation of γH2AX (Figure 4C and Figure S4C). p-Ser^216^ CDC25C kinase was also more significantly activated in siNEDD9-depleted cell lines versus SCR controls after 6 h of UV exposure (Figure 4D and Figure S4D), suggesting that UV-irradiated cells with depleted NEDD9 would be more likely to enter mitosis to a greater extent than SCR control transfected cells, potentially resulting in mitotic catastrophe and enhanced cell death. To test this hypothesis, we assessed the long term-effects of UV irradiation on the proliferative capacity of NEDD9-depleted NSCLC cells. After the transfection of each of the cell line models with SCR or siNEDD9 siRNAs and UV-irradiation (5 J/cm^2^ for murine cells, 10 J/cm^2^ for human cells) for 48 h, the post-transfection cells were seeded in a low density (500–1000 cell per well of 6 well plate) and the formation of colonies was assessed 12 days after exposure (Figure 4E and Figure S4E). Strikingly, in four out of five cell line models, UV-irradiation after siNEDD9 depletion resulted in the significantly decreased viability of irradiated cells vs. the non-irradiated cells, with an even more profound effect versus the SCR treatment and irradiation in two human and one murine NSCLC cell lines, with 10–50% greater reduction in colony formation in siNEDD9 cells. This very striking result implies that the depletion of NEDD9 and UV-irradiation is capable of imposing an insuperable cell cycle defect in NSCLC cells that requires >72 h to fully manifest, resulting in increased sensitivity to UV irradiation in the long term. 

### 3.5. Decreased NEDD9 Levels Precede Human NSCLC Metastasis and Are Associated with Decreased Survival

Given the observed effects of NEDD9 depletion on the mesenchymal phenotype and proliferation in NSCLC cell line models, we next aimed to assess if changes in the NEDD9 levels potentially correlate with NSCLC progression and patients’ survival. To gain insight into this, we performed survival and correlative analyses using the datasets available from the cBioportal [27], containing RNAseq data from NSCLC tumors, and benchmarked this analysis to our dataset of IHC-stained NSCLC patient samples to assess the potential consequences of alterations in NEDD9 levels on both the RNA and protein levels. 

**Figure 5:**
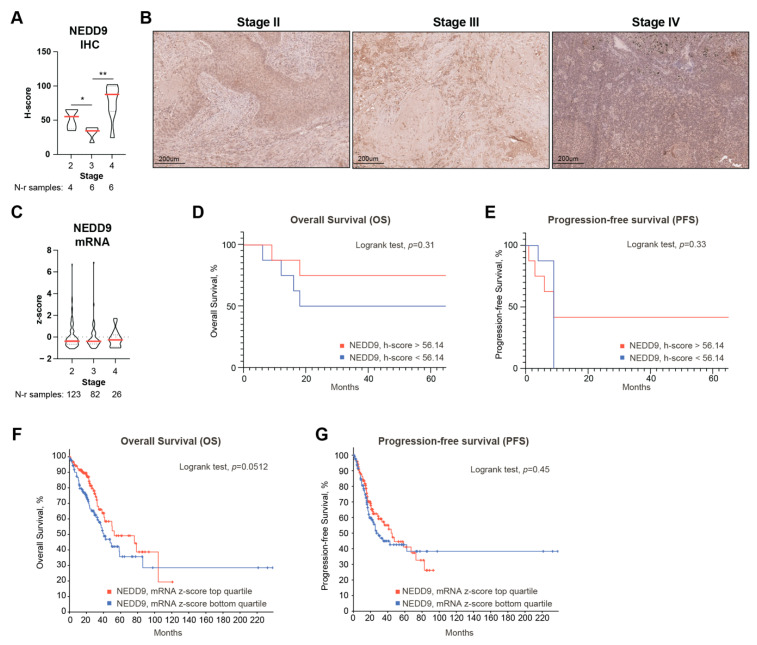
Lower NEDD9 levels are associated with worse prognosis for NSCLC patients: (**A**,**B**) NEDD9 IHC-based H-score quantification (**A**) and representative histopathological images (**B**) segregated by stage, assessed in cohort of 16 primary NSCLC tumor patient samples. Scale bar: 200 um. (**C**) NEDD9 mRNA levels (RNA-seq data) in 566 tumor tissue samples TCGA dataset. (**D**,**E**) Kaplan–Meier analysis of overall (**D**) and progression-free (**E**) survival based on low and high levels of NEDD9 protein expression (dichotomized by median H-score—56.14) in cohort of 16 IHC samples. (**F**,**G**) Kaplan–Meier analysis of overall survival (**F**) and progression-free survival (**G**) in TCGA RNA-seq datataset, with NEDD9 expression dichotomized on top vs. bottom expression z-score quartiles in 566 samples. *, *p* ≤ 0.05; **, *p* ≤ 0.01 for pairwise comparisons.

Using a quantitative immunohistochemical (IHC) approach, we assessed the relationship between the NEDD9 expression levels and the stage of the disease in the cohort of primary NSCLC tumor samples (*n* = 16) (Figure 5A,B). Even with a limited number of samples, we detected a statistically significant decrease in NEDD9 protein expression levels in stage III samples compared to stage II and stage IV samples, which (the latter) is associated with the presence of distant metastases. The analysis of RNAseq data in the TCGA dataset (*n* = 566) did not reveal any significant variations in NEDD9 mRNA expression across different stages (Figure 5C), implying that NEDD9 level changes are altered on the protein level only. For each cohort, we evaluated the overall survival (OS) and the progression-free survival (PFS) associated with NEDD9 expression in the primary tumors. For the IHC cohort, we stratified the level of NEDD9 expression as high or low based on the median H-score of 56.16, while for the TCGA cohort, we used top vs. bottom expression quartiles dichotomization. Based on this analysis, a positive correlative trend between the decreased expression of NEDD9 and the decrease in the overall (OS) and progression-free survival (PFS) was revealed in the IHC cohort (Figure 5D,E). The analysis of the TCGA dataset harboring the RNA seq data revealed a statistically borderline correlation (*p* = 0.0512) between low NEDD9 expression and decreased OS (Figure 5F); however, NEDD9 mRNA expression levels did not correlate with PFS (Figure 5G). These data suggest that the decreased expression of NEDD9, both at the protein and RNA levels, has a trend to positively correlate with a negative prognosis in NSCLC; however, future studies in an extended patient samples cohort are strongly warranted to obtain a more robust statistical data.

## 4. Discussion

An altered scaffolding function of NEDD9 protein, which is critical in sustaining numerous pro-oncogenic and tumor-suppressing pathways that are extensively cross-regulated, has been extensively reported in the literature in several cancer settings, including NSCLC [24,43]. Although NEDD9 has been much studied in the context of tumor initiation and progression [22,23,24], ciliary signaling [21], integrin signaling, and migration [15,16,44], little is known about the role of this protein in DNA damage response. The latter becomes particularly relevant in the context of lung cancer, with recent publications reporting that *KRAS*-mutant NSCLC tumors rely on non-canonical integrin/FAK [45] and ATM-related signaling [8] to drive cell proliferation, epithelial-to-mesenchymal transition, invasion, and resistance to conventional DNA damaging therapies. 

Our data demonstrate that NEDD9 is capable of restraining the DNA damage response to platinating (such as cisplatin) and other cross-linking agents, such as ultraviolet irradiation, by restraining ATM and ERCC4 kinases signaling in multiple lung cancer cell models, which defines *NEDD9* as a candidate modifier gene in this context. The loss of this suppression results in the activation of the processes of EMT, leading to more mesenchymal and more aggressive phenotypes associated with enhanced NSCLC cellular proliferation in vitro and the decreased survival of patients. The elevation of ERCC4 signaling upon the transient depletion of NEDD9 levels resulted in increased sensitivity to UV irradiation in our models, suggesting that assessing and altering NEDD9 levels in established tumors can potentially be exploited as a novel response prediction marker or modulator for irradiation therapies. 

Several reasons for the results obtained in this study can be proposed. First, NEDD9 as a member of CAS-family proteins, mechanistically interacts with numerous SRC-family kinases to phosphorylate various downstream proteins, most of which have been studied in the context of metastatic disease [46]. SRC kinase functions as a signal transducer from the surface sensors and adhesion mediators, such as integrins, to transform these stimuli into biochemical outputs executing cellular response and adaptation [47]. Recent studies demonstrated that integrins are capable of regulating DNA damage response and sensitizing *KRAS*-mutant NSCLC cells to irradiation [33]. Our observations of the specific signaling consequences of the NEDD9 depletion involved in UV-induced DDR, resulting in increased sensitivity to UV irradiation, align with the published literature [33] and undoubtedly merit further investigation. 

Second, the processes of autophagy and EMT can both contribute to cisplatin resistance in a lung cancer setting [48]. Recently published work demonstrated that elevated non-canonical ATM signaling is capable of inducing EMT in cisplatin-resistant NSCLC cell lines by upregulating JAK/STAT3 signaling and enhancing PD-L1 expression [8]. Another work demonstrated that the inhibition of autophagy sensitizes small cell lung cancer cells to cisplatin treatments, in part by downregulating NEDD9 expression [49]. These observations are compatible with our model in which NEDD9 depletion is capable of promoting EMT through a mechanism that requires enhanced ATM kinase activity in cells. In addition, our results shed light on the potential therapeutical benefit of targeting NEDD9, which can be exploited to increase sensitivity to other therapies of value for cisplatin-resistant NSCLC, including PD-1/PD-L1 targeting agents, via mechanisms mediated by NEDD9-ATM-related signaling. By defining NEDD9 as a regulator of ATM activation, our data suggest that NEDD9 expression may also be a potential biomarker for the in vivo response of immune checkpoint inhibitors (ICIs). Future studies evaluating these caveats in extended patient cohorts are strongly warranted.

Earlier studies investigating effects of elevated NEDD9 expression on tumors’ invasiveness and metastasis [46] subsequently concentrated research efforts on elucidating the role of upregulated NEDD9 in tumor progression. Our study demonstrates that elevated NEDD9 expression in late-stage tumors is not preceded by a gradual increase in expression from earlier stages of disease progression but rather by a drop in expression prior to the invasion and metastasis stage, which can be explained by progressive changes in the oncogenic signaling networks before NSCLC cells tolerate NEDD9 upregulation. 

With the common treatment challenges of overcoming the resistance to DNA damaging therapies and given that NEDD9 expression can affect the sensitivity to UV irradiation and regulate ATM-related non-canonical EMT signaling, this protein merits further investigation as a potential biomarker for treatment response in an NSCLC setting.

## 5. Conclusions

In this study, we identified a novel role for the scaffolding protein NEDD9 in regulating the ATM–CHK2 DNA damage response signaling pathway in a non-small cell lung cancer (NSCLC) setting. Based on this work, we found that NEDD9 restrains ATM-CHK2 signaling in NSCLC cells, with low NEDD9 levels associated with enhanced DNA damage, the upregulation of the ATM-CHK2 pathway, a shift towards a more mesenchymal phenotype, and an elevated sensitivity to UV irradiation. The immunohistochemical analysis of the cohort of NSCLC patient samples revealed an association between reduced NEDD9 protein expression and a decrease in overall survival (OS) of the patients. These data suggest that NEDD9 has significant merit as a potential biomarker for the response of DNA damaging therapies in an NSCLC setting.

## Data Availability

The data are contained within the article.

## Supplementary Materials

The following supporting information can be downloaded at: https://www.mdpi.com/article/10.3390/cancers14102517/s1, Table S1: Detailed clinical characteristics of NSCLC specimens; Figure S1: Depletion of Nedd9 affects clonogenic capacity of murine NSCLC cells; Figure S2: Analysis of alterations in cell cycle in NEDD9 depleted NSCLC cells; Figure S3. Cell migration (scratch wound-healing assay); Figure S4. NEDD9 depletion is associated with increased sensitivity to UV-induced dsDNA breaks; Supplementary Data: Original images of Western Blot.
