# Supplementary material for: NEDD9 Restrains dsDNA Damage Response during Non-Small Cell Lung Cancer (NSCLC) Progression
Source: Cancers (Basel). 2022 May 20;14(10):2517. doi: 10.3390/cancers14102517 (PMC9139181; doi:10.3390/cancers14102517)
Supplement: Supplementary file 1 [file cancers-14-02517-s001.zip › cancers-1675179-supplementary.pdf]

For the best experience, open this PDF portfolio in Acrobat X or Adobe Reader X, or later.

Get Adobe Reader Now!
